# The patatin-like phospholipase *Pf*PNPLA2 is involved in the mitochondrial degradation of phosphatidylglycerol during *Plasmodium falciparum* blood stage development

**DOI:** 10.3389/fcimb.2023.997245

**Published:** 2023-11-20

**Authors:** Serena Shunmugam, Nyamekye Quansah, Ansgar Flammersfeld, Md Muzahidul Islam, Juliane Sassmannshausen, Sandra Bennink, Yoshiki Yamaryo-Botté, Gabriele Pradel, Cyrille Y. Botté

**Affiliations:** ^1^ Apicolipid Team, Institute for Avanced Biosciences, Centre National pour la Recherche Scientifique (CNRS) UMR5309, Institut National de la Santé et de la Recherche Médicale, Université Grenoble Alpes, Grenoble, France; ^2^ Division of Cellular and Applied Infection Biology, Institute of Zoology, RWTH Aachen University, Aachen, Germany; ^3^ International Centre for Genetic Engineering and Biotechnology, New Delhi, India

**Keywords:** Apicomplexa, malaria, lipid, phospholipase, metabolism, lipidomic, mitochondria

## Abstract

*Plasmodium falciparum* is an Apicomplexa responsible for human malaria, a major disease causing more than ½ million deaths every year, against which there is no fully efficient vaccine. The current rapid emergence of drug resistances emphasizes the need to identify novel drug targets. Increasing evidences show that lipid synthesis and trafficking are essential for parasite survival and pathogenesis, and that these pathways represent potential points of attack. Large amounts of phospholipids are needed for the generation of membrane compartments for newly divided parasites in the host cell. Parasite membrane homeostasis is achieved by an essential combination of parasite *de novo* lipid synthesis/recycling and massive host lipid scavenging. Latest data suggest that the mobilization and channeling of lipid resources is key for asexual parasite survival within the host red blood cell, but the molecular actors allowing lipid acquisition are poorly characterized. Enzymes remodeling lipids such as phospholipases are likely involved in these mechanisms. *P. falciparum* possesses an unusually large set of phospholipases, whose functions are largely unknown. Here we focused on the putative patatin-like phospholipase *Pf*PNPLA2, for which we generated an glmS-inducible knockdown line and investigated its role during blood stages malaria. Disruption of the mitochondrial *Pf*PNPLA2 in the asexual blood stages affected mitochondrial morphology and further induced a significant defect in parasite replication and survival, in particular under low host lipid availability. Lipidomic analyses revealed that *Pf*PNPLA2 specifically degrades the parasite membrane lipid phosphatidylglycerol to generate lysobisphosphatidic acid. *Pf*PNPLA2 knockdown further resulted in an increased host lipid scavenging accumulating in the form of storage lipids and free fatty acids. These results suggest that *Pf*PNPLA2 is involved in the recycling of parasite phosphatidylglycerol to sustain optimal intraerythrocytic development when the host resources are scarce. This work strengthens our understanding of the complex lipid homeostasis pathways to acquire lipids and allow asexual parasite survival.

## Introduction

There are 241 million cases of malaria per year worldwide, of which 627 000 result in death, mostly children in Sub-Saharan Africa and South-East Asia. Malaria control has struggled to improve, due to rapid emergence of drug resistant strains, and has been exacerbated due to the COVID19 pandemic. The global and rapid emergence of drug resistant parasite strains has made the search for novel drug targets even more urgent and pertinent in recent years (World Health Organization, 2020). Malaria is caused by an obligate intracellular parasite protist called *Plasmodium* spp., which belongs to the phylum Apicomplexa. The clinical symptoms and lethal outcome of the disease are due to the asexual intracellular development of the parasite within the host’s red blood cells (RBCs). These parasites must develop and divide within erythrocytes. Understanding the metabolic interactions by which the parasite acquires essential host nutrients, especially during blood stages, is crucial in identifying key pathways that could be targeted as potential drug targets.

The intraerythrocytic phase of the life cycle comprises the growth of the ring to the trophozoite stage and the subsequent formation of merozoites by the multinucleated schizont and hence is a period of intense membrane biogenesis. Current evidence shows that large amounts of lipids are needed for survival and replication of the parasite during the intense asexual intra-erythrocytic replication cycle. Throughout this part of the parasite life cycle, lipids are essential for membrane biogenesis, assembly and division of daughter cells and their intracellular compartments/organelles (Botté et al., 2013; Amiar et al., 2016; Amiar et al., 2020; Dass et al., 2021; Shunmugam et al., 2022), but also for signalling events (Bullen et al., 2016), and for storage, essential to mobilize and provide lipids specifically when parasite division occurs (Dass et al., 2021; Sheokand et al., 2023). This translates into a ~500-800% lipid increase in infected RBCs compared to uninfected ones, involving a dedicated metabolic program of synthesis, acquisition, trafficking, remodeling and trafficking to maintain lipid homeostasis in each of the parasite compartments (Déchamps et al., 2010; Ramakrishnan et al., 2012; Tran et al., 2016; Kilian et al., 2018).

During intraerythrocytic replication, the parasite is metabolically very active and has to acquire most of its resources from the host, especially in the trophozoite and schizont stages, to allow the formation and division of future daughter merozoites. Host lipid acquisition is key for this part of the parasite development (Dechamps et al., 2010; Botté et al., 2013), and most enzymes capable of manipulating lipids have their peak of expression in these stages (Dechamps et al., 2010; PlasmoDB, 2022). Blood stage schizogony hence relies on the availability of host nutrients, particularly of lipids. Indeed, recent studies demonstrated that lysophosphatidylcholine (LPC) present in the host serum is a key limiting factor allowing maintenance of asexual blood stage division, and lack of LPC induces sexual commitment, resulting in the formation of gametocytes to prepare for human-to-mosquito transmission (Brancucci et al., 2017; Wein et al., 2018). LPC provides the key building blocks for active membrane biogenesis, i.e., choline for the synthesis of phosphatidylcholine (PC, i.e. the major phospholipid (PL) class in the parasite membrane), and fatty acids (FAs, i.e. integral components of all phospholipids). In this context, a plasmodial glycerophosphodiesterase, PfGDPD, has recently been identified that is linked to PC biosynthesis by releasing choline from glycerophosphocholine (Ramaprasad et al., 2023).

The majority of FAs required by the parasite for blood stage growth are scavenged from the host, in particular palmitic acid (C16:0), and oleic acid (C18:1), which are essential for parasite survival (Mi-Ichi et al., 2006; Nolan et al., 2017; Dass et al., 2021). Under limiting circumstances, FAs can also be synthesized *de novo* by the parasite via the prokaryotic type II FA synthase FASII, that is present in the apicoplast, a relict, non-photosynthetic plastid of the parasite. The FASII pathway has been shown to provide specific FAs, and is particularly essential during liver-stage schizogony and sporozoite formation (Mi-Ichi et al., 2006; Mi-Ichi et al., 2007; van Schaijk et al., 2014). However, the activity of the apicoplast FASII is also directly influenced by the levels of host nutrients during asexual blood stages, as it can be upregulated, thus becoming essential, in conditions where host nutrients and scavenging are scarce (Mi-Ichi et al., 2006; Mi-Ichi et al., 2007; Yu et al., 2008; Vaughan et al., 2009).

Membrane biogenesis during erythrocytic replication requires the action of phospholipases (PLs), which are classified into four groups, A, B, C and D corresponding to the hydrolysis activity, and more than 20 putative phospholipases have been identified in *P. falciparum* (Flammersfeld et al., 2018; Burda et al., 2023). Only few of these were linked so far to the erythrocytic replication phase of the malaria parasite. Probably the best-studied plasmodial phospholipase is PI-PLC (PF3D7_1013500), which was shown to be involved in calcium-dependent signaling pathways leading to merozoite invasion of RBCs. Early studies showed that chemical inhibition of PI-PLC prevents calcium-mediated signaling and thus activation of the plasmodial protein kinase B by calmodulin (Vaid and Sharma, 2006; Vaid et al., 2008; Singh et al., 2010; Raabe et al., 2011). A new study demonstrated that gene deletion of PI-PLC affected schizont maturation by impairing phosphoinositide homeostasis (Burda et al., 2023). In addition, another recent study has assigned a role of the ecithin:cholesterol acyltransferase LCAT (PF3D7_0629300) to the erythrocytic phase of *P. falciparum*. Conditional ablation of LCAT resulted in abnormal egress and a reduced replication rate by altering the levels of several phosphatidylserine and acylphosphatidylglycerol species (Ramaprasad et al., 2023).

Four of the *P. falciparum* PLs were previously identified as putative patatin-like PLs (PNPLAs), which typically exhibit a catalytic Ser-Asp dyad with the serine residue being embedded within the conserved penta-peptide Gly-Xaa-Ser-Xaa-Gly (GXSXG). PNPLAs show PLA_2_ activity and are able to generate LPLs and FAs (Kienesberger et al., 2009; Ramanadham et al., 2015; Wilson and Knoll, 2018). Two previous studies aimed at unveiling the role of one of the PNPLAs in the *P. falciparum* blood stages (PF3D7_0209100), coming to contradictory results. One study reported that deficiency of the PL (here termed PNPLA1) results in upregulated PL levels as well as impaired gametocyte induction both under normal growth conditions and in PL precursor-deficient medium, suggesting that the deregulated PL levels in the asexual blood stages render these less sensitive to modulators of gametocytogenesis (Flammersfeld et al., 2020). The other study assigned the PL (here termed PATPL1) to gametogenesis, since gene deletion was linked to reduced egress, and exflagellation of gametocytes (Singh et al., 2019).

Here we functionally characterized one further patatin-like PL, termed *Pf*PNPLA2 (PF3D7_1358000). We reveal *Pf*PNPLA2 to be active in the parasite mitochondria using lipidomics approaches. We also demonstrate that *Pf*PNPLA2 has a critical role during low host nutrient condition, when the protein becomes essential for asexual blood stage survival, especially at the schizont-to-ring transition. In addition, we show that *Pf*PNPLA2 degrades phosphatidylglycerol (PG) to allow the synthesis of lysobisphosphatidic acid (LBPA). This likely allows the generation of FA to boost synthesis of membrane PLs, potentially facilitating parasite division and progeny formation. Our work further emphasizes the importance of mobilization and channeling of lipid resources, here PG degradation/recycling, for asexual blood stage parasite development within the human host RBC.

## Materials and methods

### Primary antibodies

In this study, the following primary antibodies and antisera were used: Rabbit anti-HA antibody (Sigma-Aldrich); rat anti-HA antibody (Roche); mouse anti-GFP antibody (Sigma-Aldrich); rabbit polyclonal antisera against *Pf*s230 (Biogenes), *Pf*s25 (ATCC), *Pf*s16, AMA1 (Boes et al., 2015); mouse polyclonal antisera against falcilysin (Weißbach et al., 2017), *Pf*39 (Simon et al., 2009) and actin I (Ngwa et al., 2013). The generation of polyclonal mouse antisera against *Pf*PNPLA2, 3 and 4 is described below.

### Generation of mouse antisera

Recombinant proteins of *Pf*PNPLA2 (spanning aa 571-998), *Pf*PNPLA3 (spanning aa 589-763) and *Pf*PNLPA4 (spanning aa 2-187) were expressed for the generation of antisera. *Pf*PNPLA2 was expressed with an N-terminal maltose binding protein (MBP)-tag using the expression vector pIH902. Cloning was mediated by the addition of the restriction sites EcoRI and PstI (for primer sequences see **Supplementary Table 1**). *Pf*PNPLA3 was expressed with a C-terminal His-tag using pET28c (+) and cloning was mediated by the addition of NcoI/XhoI restriction sites. *Pf*PNPLA4 was expressed as a fusion protein with an N-terminal MBP-tag using pMalc5x. Cloning was mediated by XmnI/PstI restriction sites. Recombinant fusion proteins were expressed using *E. coli* BL21 (DE3) RIL (Stratagene) and purified via affinity chromatography using amylose resin (New England Biolabs) for the MBP-tagged proteins and complete™ His-tag purification resin (Sigma-Aldrich) for the His-tagged proteins according to the manufacturers’ protocols. After PBS buffer exchange via filter centrifugation using Amicon Ultra 15 (Sigma-Aldrich), protein concentrations were determined by Bradford assay. 100 µg recombinant proteins emulsified in Freund’s incomplete adjuvant (Sigma-Aldrich) were injected into 6 weeks-old NMRI mice (Charles River Laboratories) via subcutaneous injection. After 4 weeks, a boost with 50 µg recombinant proteins was injected. Ten days after the boost, mice were anesthetized by intraperitoneal injection of a ketamine/xylazine mixture (Sigma-Aldrich) according to the manufacturer’s protocol and immune sera were collected via heart puncture. Sera were pooled from three mice immunized with the same antigen. Experiments for the generation of antisera in mice were approved by the animal welfare committees of the District Council of Cologne, Germany (ref. no. 84-02.05.30.12.097 TVA).

### Generation of the *Pf*PNPLA2- and *Pf*PNPLA4-KD parasite lines

*Pf*PNPLA2- and *Pf*PNPLA4-KD parasite lines were generated via single cross-over homologous recombination using the pARL-HA-glmS vector (Flammersfeld et al., 2020). A 1015-bp gene fragment homologous to the 3’ end of the gene coding for *Pf*PNPLA2 and a 840-bp gene fragment of the gene coding for *Pf*PNPLA4 (excluding the stop codons) were amplified using corresponding primers (for primer sequences see **Supplementary Table 1**). Ligation of the inserts with the vector backbone was mediated by NotI/AvrII restriction sites. A synchronized wildtype (WT) NF54 culture containing 5% ring stages was loaded with 100 µg vector in transfection buffer via electroporation as described. Parasites carrying the vector were selected by the addition of WR99210 (Jacobus Pharmaceuticals) in a final concentration of 4 nM and successful integration of the vector was confirmed by diagnostic PCR using (1) 5’-integration primer, (2) 3’-integration primer, (3) pARL-HA-glmS forward primer and (4) pARL-HA-glmS reverse primer (for primer location see **Supplementary Figure 2A**; for primer sequences see **Supplementary Table 1**).

### Diagnostic RT-PCR

Total RNA was isolated from synchronous ring, trophozoite and schizont cultures, as well as from Percoll-gradient enriched immature, mature and activated gametocytes as described before (Flammersfeld et al., 2020). RNA preparation and subsequent cDNA synthesis were performed as described (Flammersfeld et al., 2020). Transcripts for *Pf*PNPLA2 (250 bp), *Pf*PNPLA3 (250 bp) and *Pf*PNPLA4 (250 bp) were amplified in 25 cycles using the corresponding primers (for primer sequences see **Supplementary Table 1**). To confirm purity of the asexual blood stage and gametocyte samples, transcript amplifications of *ama1* (407 bp) and of *ccp2* (286 bp) were performed using the corresponding primers. Amplification of the housekeeping gene *aldolase* (378 bp) was used as loading control.

### Indirect immunofluorescence assay and live cell imaging

Synchronized asexual cultures of a wildtype (3D7) strain and the PNPLA2-iKD line were maintained as previously described (Trager and Jensen, 1976; Trager et al., 1980). Parasite cultures (200 µl of compact infected RBCs) were collected by centrifugation (10 min at 1200 rpm at room temperature). Parasites were fixed with 4% paraformaldehyde and 0.0075% glutaraldehyde for 30 min at room temperature. Cells were centrifuged at 3000 rpm for 2 mins and the supernatant was discarded. The sample was then washed twice with PBS and the supernatant removed. The pellet was resuspended gently in 1 ml of 4% FBS (fetal bovine serum) in PBS and spun again as previously. Cells were permeabilized using 0.1% Triton X-100 in PBS for 10 min. Cells were pelleted and the sample was washed thrice with PBS. The pellet was saturated with 4% FBS in PBS and incubated at 4°C for 1 h. There after the primary antibodies of HA-tag (Roche) or anti-PNPLA2/PNPLA3/PNPLA4 (homemade) LBPA (Tebu bio) were added at dilutions of 1:500 or 1:50, respectively. The antibodies were then washed away thrice with PBS. The secondary antibodies (AlexaFluor488/546 anti-rat/mouse) were used at 1:1000 dilutions in FBS and incubated for 1 h. Hoechst stain was used at 1:25000 dilution for 20 mins and finally samples were washed with PBS thrice. Pellets were resuspended in 150 µl of PBS and then gently mixed with the same amount of Fluorogel. 15 µl of each sample was pipetted on a microscope slide and overlayed with a square coverslip. Samples were allowed to solidify in the dark at 37°C for 1 h. Specimens were examined with an Axio Imager; ZEISS, ×100 magnification. Images were treated using ImageJ software. Indirect immunofluorescence images were obtained for gametocytes as previously described (Flammersfeld et al., 2020).

### Assessment of parasite mitochondrial morphology

Mixed asexual blood stages cultures of a wildtype (3D7) strain and the PNPLA2-iKD line were maintained as previously described (Trager and Jensen, 1976; Trager et al., 1980). Parasite cultures (200 µl of compact infected RBCs) were collected by centrifugation (10 min at 1200 rpm at room temperature). Cultures were resuspended in media with or without 2.5 mM GlcN and incubated for 48 h. These cultures were then collected by centrifugation (10 min at 1200 rpm at room temperature) and resuspended in prewarmed RPMI containing 100 µM Mitotracker™ and incubated for 45 min. After the incubation, specimens were examined with an Axio Imager; ZEISS, ×100 magnification. Images were treated using ImageJ software.

### Western blotting

Western blotting was performed as previously described (Flammersfeld et al., 2020). Briefly, tightly sorbitol-synchronized schizont stage parasite samples underwent saponin lysis to remove red blood cell membrane (Hall et al., 1983). The sample was then resuspended in a lysis buffer containing an anti-protease cocktail (0.5% Triton X- 100, 4% w/v SDS, 0.5× PBS). SDS-PAGE buffer was added, and samples were denatured at 95°C for 10 mins. Equal sample volumes were then separated by SDS-PAGE and proteins were transferred onto a Hybond ECL nitrocellulose membrane (Amersham Biosciences) according to the manufacturer’s protocol. The blot was blocked using Tris-buffered saline containing 5% w/v skim milk, pH 7.5, followed by overnight incubation at 4°C with polyclonal mouse immune sera specific for *Pf*39 (dilution 1:5,000) or using polyclonal rabbit immune sera specific to anti-HA (1:1,000). After washing, membranes were incubated with the respective alkaline phosphatase-conjugated secondary antibody (Sigma-Aldrich) for 1 h at RT and developed in a solution of nitroblue tetrazolium chloride (NBT) and 5-brom-4-chlor-3-indoxylphosphate (BCIP; Sigma-Aldrich) for 5-30 min at RT. Blots were scanned and processed using the Adobe Photoshop CS software. Band intensities were measured using the ImageJ programme version 1.51f.

### Asexual blood stage replication assay

To analyze asexual blood stage replication and length of the intraerythrocytic replication cycle in the PNPLA2-HA-iKD parasite line, experiments were set-up as follows: Expression of PNPLA2 was knocked-down in transgenic PNPLA2-HA-iKD parasite line by treatment of the culture with GlcN (D-(+)-glucosamine hydrochloride; Sigma Aldrich) at a final concentration of 2.5 mM. GlcN treatment started one cycle before the experimental set-up. The GlcN-treated culture was compared to the untreated culture cultivated in normal cell culture medium. This assay was also done in minimal media only including RPMI, glucose, hypoxanthine (with 1 M NaOH), FA-free bovine serum albumin (60 µM) and FFA C16:0 (30 µM) and C18:1 (30 µM). To exclude an unspecific effect of GlcN, the parental WT line was used as control. For each assay, three experiments were performed, each in triplicate.

### Gametocyte development assay

To induce knockdown of *Pf*PNPLA2, a synchronized parasite culture of the *Pf* PNPLA2-HA-iKD line was treated with 2.5 mM GlcN for 72 h. Gametocytogenesis was subsequently induced with lysed blood cells while the GlcN treatment was continued. The gametocytemia was evaluated by Giemsa smears over a period of six days. Untreated *Pf* PNPLA2-HA-iKD cultures were used as controls.

### Lipidomics analysis

Lipidomics analysis was performed on 3-4 independent cell harvests of the PNPLA2-HA-iKD line. Highly synchronous parasite cultures grown for at least 48 hours with or without glucosamine (ring/trophozoite or schizont stage) were harvested by transferring the cultures to pre-warmed 50-ml tubes and the total amount of parasites was determined by Giemsa staining and via counting on a hemocytometer. The cultures were metabolically quenched via rapid cooling to 0°C by suspending the tube over a dry ice/100% ethanol slurry mix, while continually stirring the solution. Following quenching, parasites were constantly kept at 4°C and liberated from the enveloping RBCs by mild saponin treatment with 0.15% w/v saponin/0.1% w/v BSA PBS for 5 min. After three washing steps with 1 ml ice-cold PBS followed by centrifugation, the parasite pellet was subjected to lipid extraction using chloroform and methanol (Vaid et al., 2008) in the presence of butylhydoxytluene, PC (C21:0/C21:0), C13:0 or C15:0 FA standards (Avanti). Lipid extraction, separation and analyses were all performed as previously described in Flammersfeld et al., 2020 (Flammersfeld et al., 2020). FA methyl esters were detected by GC-MS (Agilent 5977A- 7890B) according to the method described in Ramakrishnan et al. (2012) (Ramakrishnan et al., 2012) and quantified by Mass Hunter software (Agilent). Each FA was quantified according to a calibration curve generated with authentic fatty methyl ester standards.

### Statistical analysis

Data are expressed as mean ± SD. Statistical differences were determined using unpaired two-tailed Student’s t test, as indicated. Values of *p* < 0.05 were considered statistically significant. Significances were calculated using GraphPad Prism 5 and are represented in the figures as follows: ns, not significant *p* > 0.05; *p < 0.05; **p < 0.01.

## Results

### *Pf*PNPLA2 is a non-redundant parasite phospholipase that localizes to the parasite mitochondrion and is important for asexual parasite development under nutrient poor environment

**Figure 1:**
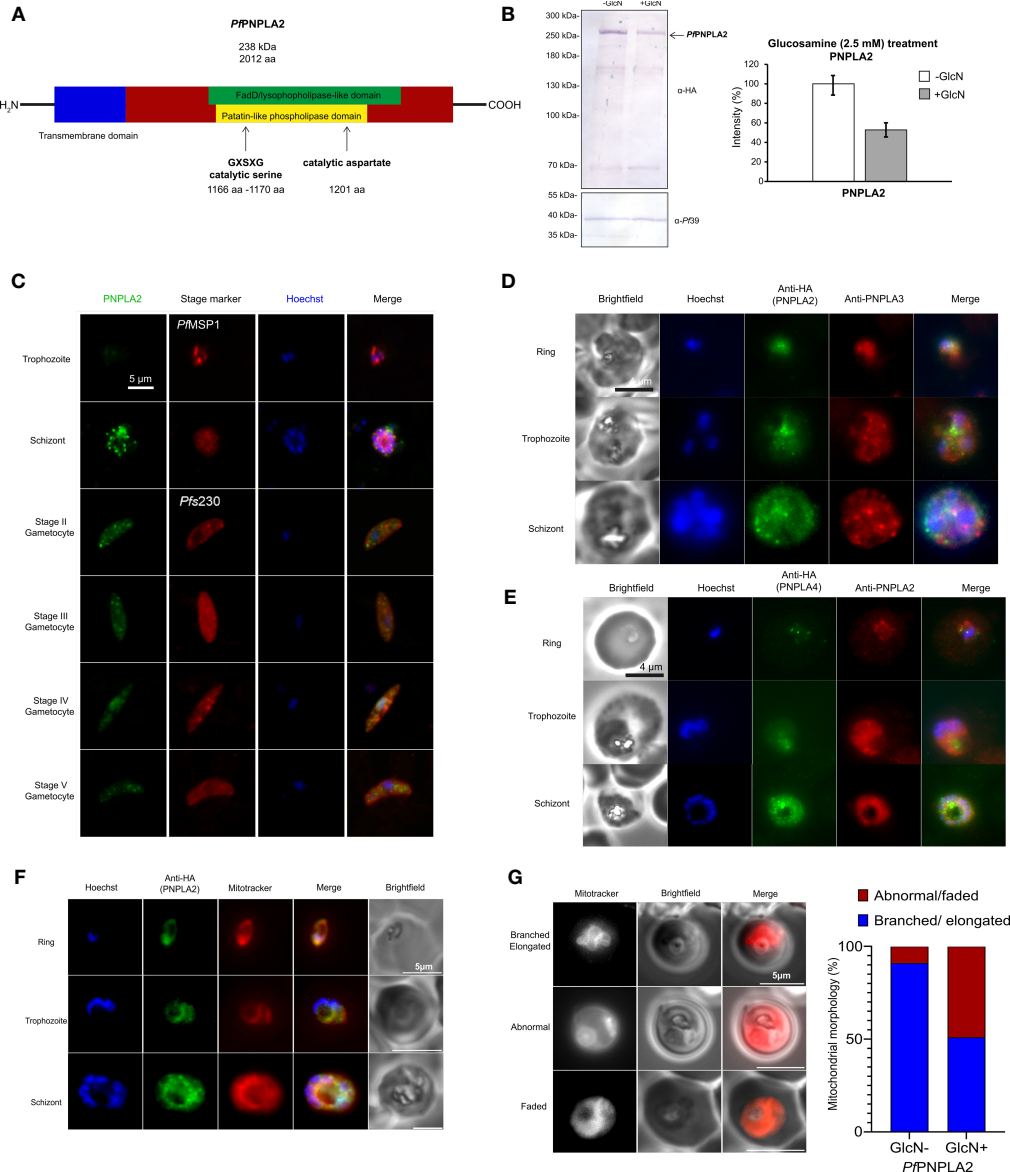
*Pf*PNPLA2 localizes to the mitochondria during different parasites stages. **(A)** A putative patatin-like phospholipase, *Pf*PNPLA2 (PF3D7_1358000) contains a FaD-like domain (green) and a patatin-like phospholipase domain (yellow) along with the GXSXG-motif and catalytic aspartate characteristic of this family of proteins. **(B)** Downregulation of *Pf*PNPLA2-HA levels. Schizont cultures of the *Pf*PNPLA2-HA-KD parasite line were treated with 2.5 mM GlcN for 72 h to determine knockdown of *Pf*PNPLA2. Lysates were immunoblotted with rat anti-HA antibody to detect *Pf*PNPLA2-HA (~240 kDa); immunoblotting with rabbit anti-*Pf*39 antisera (39 kDa) was used as loading control (bottom panel). Quantification of *Pf*PNPLA2-HA levels following knockdown. *Pf*PNPLA2-HA levels were evaluated between the GlcN-treated and untreated *Pf*PNPLA2-HA-KD parasites in three independent Western blots based on the band intensity as estimated with ImageJ software (right panel). *Pf*39 levels were used for normalization (set to 100%). **(C)** Using a mouse anti-*Pf*PNPLA2 antibody (green) for immunolabelling, *Pf*PNPLA2 is detectable in perinuclear regions in schizonts and gametocytes of stages II-V. Counter labeling of the asexual blood stages was done using rabbit anti-*Pf*MSP1 antisera and gametocytes with rabbit anti-*Pf*s230 antisera (red). The nuclei were labeled with Hoechst 33342 nuclear stain (blue). Bar; 5 µm. **(D)** *Pf*PNPLA2-HA, as detected by rat anti-HA antibody (green) does not colocalize to *Pf*PNPLA3 (red), which was immunolabelled using a mouse anti-*Pf*PNPLA3 antibody, and **(E)** *Pf*PNPLA4-HA (green), detected by rat anti-HA antibody, does not localize with *Pf*PNPLA2, immunolabelled with a mouse anti-*Pf*PNPLA2 antibody (red). **(F)** *Pf*PNPLA2 has a Mitotracker™ (red) co-localization during asexual stages using *Pf*PNPLA2-HA-glmS parasites line using an anti-HA antibody (green). **(G)** Live microscopy using Mitotracker (red), revealed that the loss of *Pf*PNPLA2 leads to degradations of the mitochondrion morphology in trophozoite stage of the parasite.

Gene PF3D7_1358000 encodes for a putative patatin-like phospholipase (Flammersfeld et al., 2018), termed *Pf*PNPLA2. The protein is predicted to be a phospholipase A2 (PLA2) with a patatin-like phospholipase domain like that of *Pf*PNPLA1 (Flammersfeld et al., 2020), and an estimated molecular weight of 238.2 kDa (**Figure 1A**). *Pf*PNPLA2 comprises a transmembrane domain at the N-terminal region of the protein. Furthermore, the characteristic GXSXG motif is present in this protein between aa 1166 and 1171, and a conserved signature catalytic aspartate found in most eukaryotic PLA2 at residue 1201 (**Figure 1A**) (Malley et al., 2018). It has been shown that all plasmodial patatin-like phospholipase homologs share the above-mentioned motif and catalytic aspartate, supporting the classification of PNPLA2 (Flammersfeld et al., 2018; Flammersfeld et al., 2020). In addition to *Pf*PNPLA1 and *Pf*PNPLA2, two more members of the *Pf*PNPLA family were previously identified, i.e. *Pf*PNPLA3 (151 kDa, encoded by gene PF3D7_0924000) and *Pf*PNPLA4 (283 kDa, encoded by gene PF3D7_0218600). *Pf*PNPLA3 comprises, in addition to the central patatin-like phospholipase domain, two N-terminal transmembrane domains (**Supplementary Figure 1A**).

Diagnostic reverse transcriptase (RT)-PCR was performed to investigate expression of *pfpnpla2, pfpnpla3 and pfpnpla4* in the *P. falciparum* blood stages. This showed the synthesis of transcripts, from all three genes, in the ring, trophozoite, and in schizonts stages. Similar transcript levels were detected in immature (stage II-IV) and mature (stage V) gametocytes as well as in gametocytes at 15’ post-activation (p.a.) (**Supplementary Figure 1B**). Transcript analysis of the housekeeping gene *aldolase* was used as a loading control, while purity of the asexual blood stage and gametocyte samples was demonstrated by amplification of transcripts for the asexual blood stage-specific gene *pfama1* (apical membrane antigen 1) (Peterson et al., 1989) and for the gametocyte-specific gene *pfccp2* (LCCL-domain protein 2) (Pradel et al., 2004). cDNA preparations lacking the reverse transcriptase were used to verify that samples were devoid of gDNA (**Supplementary Figure 1B**).

For functional characterization of *Pf*PNPLA2, we generated a conditional *Pf*PNPLA2-HA-iKD line (using the pARL-HA-glmS vector) by fusing the sequences coding for a hemagglutinin A (HA)-tag to the 3´-coding region of *pfpnpla2* (**Supplementary Figure 2A**). In the *Pf*PNPLA2-HA-iKD, the *pfpnpla2* mRNA is expressed under the control of the glmS ribozyme, which catalyzes its own cleavage in the presence of GlcN, triggering transcript degradation. Vector integration was confirmed by diagnostic PCR (**Supplementary Figure 2B**). Immunoblotting of asexual blood stage lysate with an anti-HA antibody confirmed the synthesis of an HA-tagged *Pf*PNPLA2 fusion protein in the untreated *Pf*PNPLA2-HA-iKD line, running at a molecular weight of 238 kDa (**Figure 1B**). When the disruption of *Pf* PNPLA2-HA-iKD was induced by treating asexual blood stage parasites with 2.5 mM GlcN for 72 h, PNPLA2-HA-iKD levels were reduced compared to the untreated control (**Figure 1B**).

The untreated *Pf*PNPLA2-HA-iKD line was used for in-depth protein expression analysis. Furthermore, an HA-tagged *Pf*PNPLA4 parasite line was generated (**Figure S1B**), using the above mentioned pARL-HA-glmS vector. In addition, polyclonal mouse antisera were generated against recombinant *Pf*PNPLA2, *Pf*PNPLA3 and *Pf*PNPLA4 peptides to be used for protein expression analysis. Immunofluorescence assays (IFA) revealed that *Pf*PNPLA2 is expressed throughout all asexual and sexual blood stages (**Figure 1C**). *Pf*PNPLA2 is predominantly expressed in the schizont stages and seems to localize to vesicular structures of individual merozoites formed within the parasite vacuole. *Pf*PNPLA2 could further be detected at distinct loci within the cytoplasm of gametocytes (**Figure 1C**). Closer co-localization studies revealed that *Pf*PNPLA2 does not colocalize with *Pf*PNPLA3 (or *Pf*PNPLA4), which suggests that each phospholipase has non-redundant functions (**Figures 1D, E**). Further co-localization IFA using Mitotracker™ revealed that, in the ring to schizont-stage parasites, this protein localizes to the mitochondrion (**Figure 1F**) (Mullin et al., 2006; Botté et al., 2013). The presence of *Pf*PNPLA2 at the mitochondrion led us to investigate the effect of the loss of the protein on the mitochondrion’s morphology by IFA using Mitotracker. Loss of the protein leads the mitochondrion to take on abnormal morphologies during the trophozoite and schizont stages. Instead of the usual elongated and branched form, the parasites that have lost the protein have faded or misplaced mitochondrion, among other abnormalities, also confirmed statistically significant over parasite population (**Figure 1G**). Furthermore, previous data show the protein localizing close to the apicoplast (Burda et al., 2021) leading us to the idea that this protein would have a dual function.

**Figure 2:**
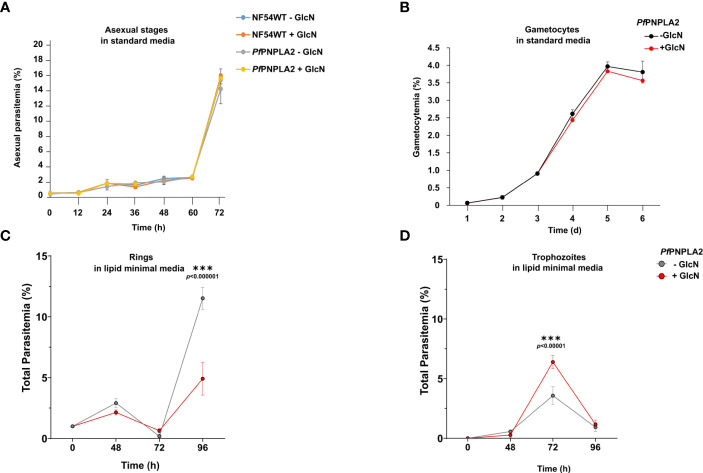
*Pf*PNPLA2 is important for blood stage replication under limiting lipid conditions. **(A)** Asexual blood-stage replication following *Pf*PNPLA2 knockdown. A highly synchronized *Pf*PNPLA2-HA-KD parasites ring stage culture was set up with a parasitemia of 0.25% and treated with 2.5 mM GlcN. The parasitemia was determined by Giemsa smears at 0 h, 12 h, 24 h, 36 h, 48 h, 60 h and 72 h. Untreated *Pf*PNPLA2-HA-KD cultures as well as GlcN-treated and untreated WT NF54 cultures were used as controls. **(B)** Gametocyte development following *Pf*PNPLA2 knockdown. A synchronized parasite culture of the *Pf*PNPLA2-HA-KD line was treated with 2.5 mM GlcN for 72 h, and gametocytogenesis was subsequently induced with lysed blood cells while the GlcN treatment was continued. The gametocytemia was evaluated by Giemsa smears over a period of six days. Untreated *Pf*PNPLA2-HA-KD cultures were used as controls. **(C, D)** In lipid minimal medium (only C18:1 and C16:0), the depletion of *Pf*PNPLA2 displays a reduction in ring stage parasites after 96 hours of glucosamine (GlcN) treatment.

To determine the importance of *Pf*PNPLA2 for parasite survival, we performed growth assays upon presence or absence (+GlcN) of the protein. Agreeing with previous phenotypic scores predicted for the protein (Nolan et al., 2017; Singh et al., 2019), knocking *Pf*PNPLA2 down in the *Pf*PNPLA2-HA-iKD line, by treatment with 2.5 mM GlcN, had no significant effect on intraerythrocytic replication during a 72 h period in regular *in vitro* culture conditions, when compared to untreated *Pf*PNPLA2-HA-iKD parasites and both treated or untreated WT NF54 parasites (**Figure 2A**). Furthermore, quantification of ring, trophozoite and schizont stages, after GlcN treatment, at different time points, did not show any major effect on parasite stage development of *Pf*PNPLA2-HA-iKD and WT NF54 parasites (**Supplementary Figure 3**), suggesting that the lack of *Pf*PNPLA2 does not delay the blood stage cycle. However, we previously showed that the host nutritional and lipid content can influence the essentiality of a protein, changing it from dispensable in normal/rich lipid conditions, to become essential under low nutritional conditions (Amiar et al., 2020). To analyze this, parasites were cultivated in minimal media (Mi-Ichi et al., 2006) containing the minimal FA requirement for *in vitro* culture, i.e. C16:0 and C18:1 (in combination to FA-free BSA). Interestingly, in these lipid-limiting conditions, the *Pf*PNPLA2-HA-iKD line, treated with GlcN, displayed a significant growth delay after 96 hours of intracellular development, linked to a decrease in ring stage parasites (**Figures 2C, D**). In sexual stage parasites, the depletion of this protein did not affect the gametocyte population (**Figure 2B**). These data may suggest that PNPLA2 is more important for asexual blood stage development than for gametocytogenesis, and the essentiality of this protein cannot be ruled out, as we were not able to attain a complete absence of the protein following knockdown. More importantly, this protein seems to be important under nutrient poor conditions and may strongly be dependent on the extracellular environment of the host.

### *Pf*PNPLA2 depletion differentially affects neutral lipid synthesis in ring and schizont stage parasites

To investigate the effect the loss of *Pf*PNPLA2 has on overall parasite lipid metabolism, we conducted global lipidomic analyses using gas-chromatography mass spectrometry on all asexual parasite stages (synchronized ring, trophozoite and schizont stages). It should be noted that despite *Pf*PNPLA2’s essentiality in a lipid poor environment, all lipidomic experiments were conducted in normal culture conditions (with Albumax) as otherwise, cultures did not yield high enough parasitemia, once the protein was knocked down.

We first sought to assess any potential defects in total lipid content and then quantified major lipid classes sustaining parasite survival, free FAs (building blocks of most lipids; FFA), diacylglycerols (major lipid precursors, DAG), PLs (major membrane lipids), and triacylglycerols (the main component of lipid storage bodies; TAG).

**Figure 3:**
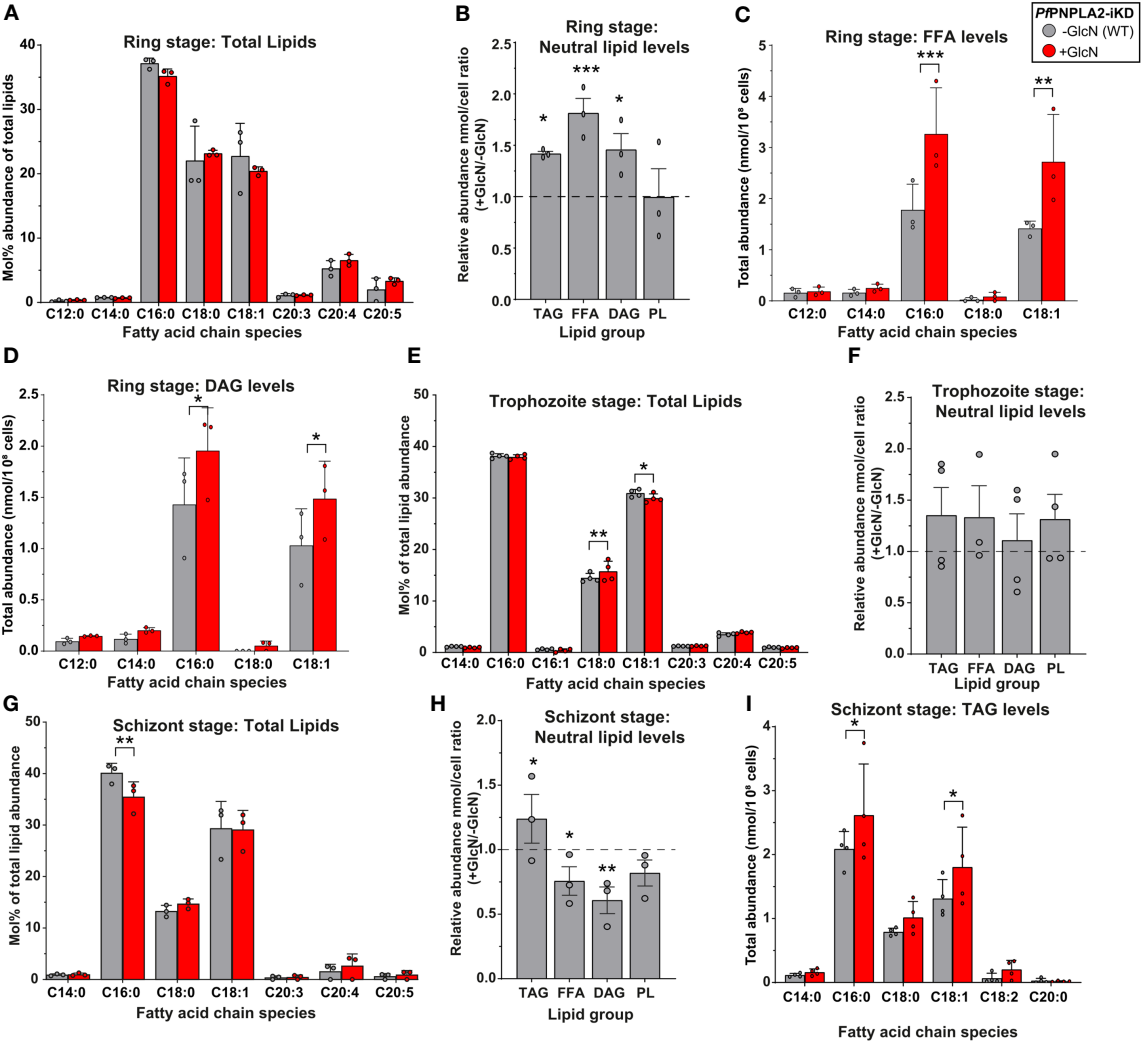
*Pf*PNPLA2 depletion influences the lipidomic profile of asexual stage parasites. **(A)** Under the depletion of *Pf*PNPLA2 (+GlcN, red) the lipid profile of ring stage parasites is not altered. **(B)** The relative neutral lipid content (TAG- triacylglycerol, FFA- free FAs, DAG-diacylglycerol) was increased when *Pf*PNPLA2 was inhibited, but the PL levels remained unaltered. **(C)** FFA and **(D)** DAG total abundance levels increased in ring stage parasites specifically for C16:0 and C18:1 fatty acyl chain species. **(E)** In trophozoite stage parasites the total lipid profile shows a significant mol% increase in C18:0 and decrease in C18:1, with no changes in **(F)** total neutral lipid abundance under the depletion of *Pf*PNPLA2. **(G)** In schizont stage parasites the depletion of the protein results in a decrease in the mol% of C16:0. **(H)** There was an increase in overall TAG levels and a decrease in FFA and DAG levels under *Pf*PNPLA2 knockdown. **(I)** TAG FA profiles were altered in schizonts under depletion of *Pf*PNPLA2 as displayed by increases in C16:0 and C18:1. The p values were calculated by Student’s t test: *p < 0.05, **p < 0.01, and ***p < 0.001.

During ring stage development, the knockdown of *Pf*PNPLA2 in the *Pf*PNPLA2-HA-KD line had no significant effect on the global FA profile of the total parasite lipid content (**Figure 3A**). However, further analyses, through separation of neutral and total PLs groups via 1D thin-layer chromatography (TLC), revealed that upon *Pf*PNPLA2 knockdown, there was significant increases in TAG, FFA and DAG, the lipid classes constituting the parasite’s neutral lipid content, but there was no impact on the total PL content of the parasite (**Figure 3B**). Further analyses to determine the FA composition of each lipid class showed that both FFA and DAG displayed significant increases in their C16:0 and C18:1 content (**Figures 3C, D**), which are predominantly scavenged from the extracellular media (Mi-Ichi et al., 2006; Mi-Ichi et al., 2007). This also correlates to our findings that growth defects following *Pf*PNPLA2 knockdown occur under low lipid content only containing C16:0 and C18:1. However, no changes could be detected in the FA profiles of TAG or total PL (**Supplementary Figures 4A, B**) under *Pf*PNPLA2 deficiency.

Knockdown of *Pf*PNPLA2 in trophozoite stage parasites exhibited limited changes in the overall FA profile of the total lipid content of the parasite, such as a significant increase in C18:0 and a significant decrease in C18:1 (**Figure 3E**). Further analyses of parasite lipid classes showed no changes in total neutral lipid content (either FFA, DAG or TAG) or PL content (**Figure 3F**). This lack of phenotype might be directly linked to a lower activity of the enzyme during trophozoite stages, evidenced by its RNA expression being the lowest during trophozoite stages (PlasmoDB, 2022).

The most severe effects were observed during the schizont stage when the protein is most expressed. First, in the total lipid FA profile, the loss of *Pf*PNPLA2 resulted in a significant decrease of the relative abundance of palmitic acid (C16:0) (**Figure 3G**). Similar to the ring stage, knockdown of *Pf*PNPLA2 in schizonts resulted in a significant increase of the storage lipid, TAG and no change to PL. However, unlike in ring stage, in schizonts, the levels of both FFA and DAG were significantly reduced (**Figure 3H**), but there were no changes in their FA species profile (**Supplementary Figures 4C, D**). The reduction of *Pf*PNPLA2 levels did not lead to any changes in the relative amount of total PL levels (**Figure 3H**). Analysis of the FA content of TAG accumulating in schizont stage in the absence of *Pf*PNPLA2 revealed a significant increase of TAG molecular species containing C16:0 and C18:1 (**Figure 3I**), as for rings, suggesting increased host scavenging. It should also be noted that there were no changes in the total lipid abundance at any stage, despite seeing clear changes in individual lipid groups (**Figures 3A, E, G**).

These data collectively suggest that *Pf*PNPLA2 is most important for FFA generation/acquisition as well as the synthesis of neutral lipids likely for storage and timely mobilization during the transition from the schizont to the ring stage parasites.

### *Pf*PNPLA2-deficiency results in an overall increase of PG and decrease of LBPA

To further investigate the putative phospholipase activity of *Pf*PNPLA2, notably determine its potential lipid substrates and products, we sought to analyze each PL class and their molecular content in schizonts by 1D or 2D HPTLC coupled to GCMS lipidomic analyses.

**Figure 4:**
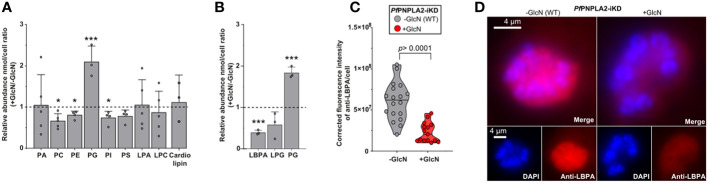
*Pf*PNPLA2 is involved in the hydrolysis of phophatidylglycerol to lysobisphosphatidic acid (n=3). **(A)** Individual phospholipid species (separated by 2D-thin layer chromatography) were analyzed by GCMS and revealed that *Pf*PNPLA2 reduction resulted in an increase in the relative abundance of (+Glucosamine (GlcN)/-GlcN) phosphatidylglycerol (PG) and a decrease in phosphatidylcholine (PC), phosphatidylethanolamine (PE), phosphatidylinositol (PI). **(B)** Closer analyses show a decrease in relative abundance of lysobisphosphatidic acid (LBPA) and not lysophosphatidylglycerol (LPG). **(C, D)** Determination of LBPA accumulation in wt parasites vs PfPNPLA2-iKD mutants by immunofluorescence assay using an anti-LBPA antibody **(D)** reveals a statistically significant decrease of LBPA in the PfPNPLA2-iKD mutant under glucosamine (GlcN) treatment (protein knock-down). The p values were calculated by Student’s t test: *p < 0.05, and ***p < 0.001.

**Figure 5:**
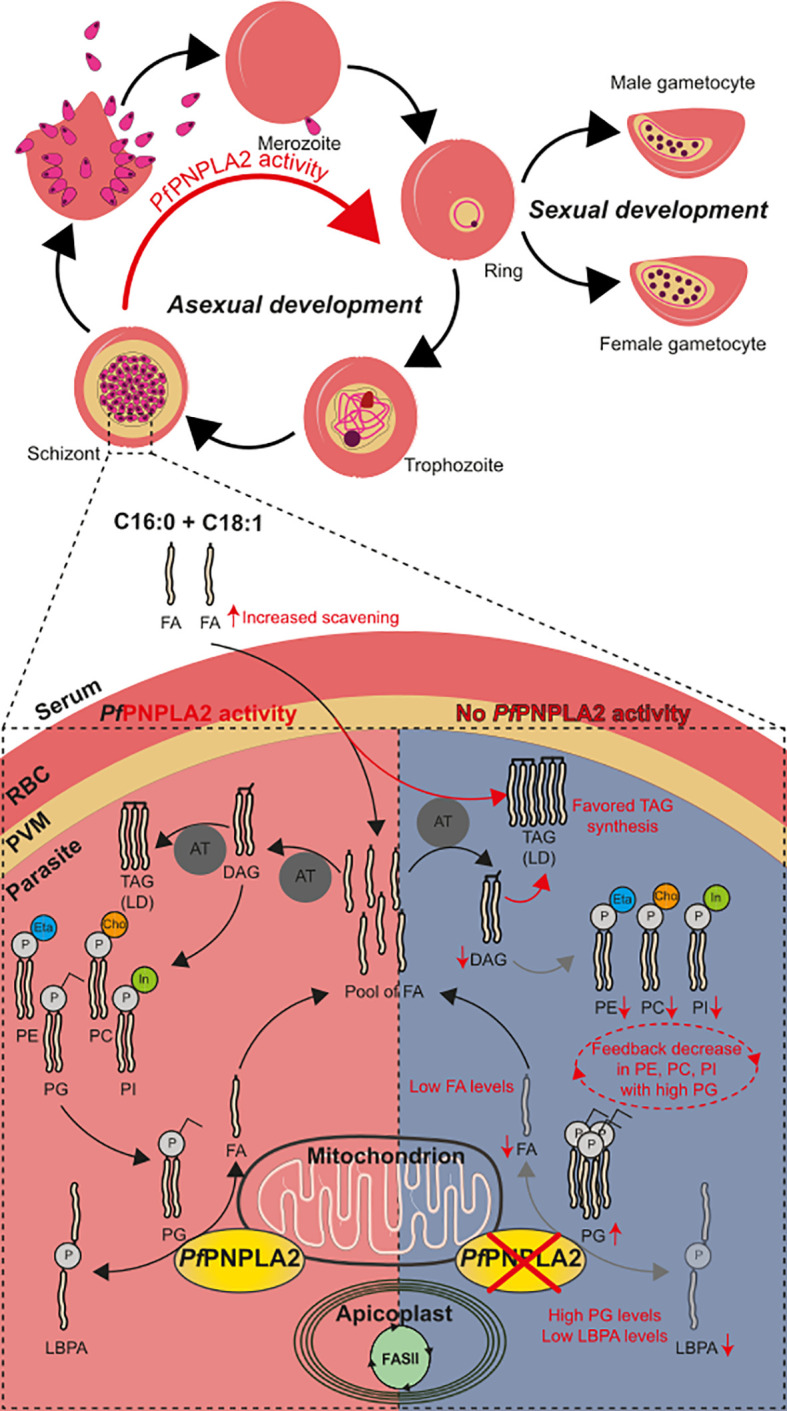
*Pf*PNPLA2 is involved parasite development via the conversion of PG to LBPA. Normal *Pf*PNPLA2 activity (during schizont and ring stages near the mitochondria) facilitates the utilization of phosphatidylglycerol (PG) into free FA and LBPA/BMP pool of FAs generated by FASII, host scavenging and this process can then contribute to lipid storage generation (triacylglycerol, TAG) and PL synthesis via the CDP-DAG pathway (PC: phosphatidylcholine, PE: phosphatidylethanolamine, PI: phosphatidylinositol). In the absence of this protein PG levels are increased with lowered levels in LBPA and FFA. We hypothesize that the lack of sufficient FFA prompts the parasite to increase host FFA scavenging of C16:0 and C18:1. This does not restabilize FFA to normal and instead TAG formation (LD: lipid droplet) is favored to escape lipotoxic death. The increase in PG has a negative feedback effect and PL synthesis (PC, PI, PE) is inhibited.

The disruption of *Pf*PNPLA2 significantly impacted parasite PL homeostasis (**Figure 4A**). There were slight, yet significant reductions in the content of phosphatidylcholine (PC), phosphatidylethanolamine (PE), phosphatidylserine (PS) and phosphatidylinositol (PI) that seemed more marginal. PLA2 usually catalyze the hydrolysis of one of the FA moieties from PL, hence generating lysoPL and FA. The levels of major lysolipids, lysophophatidylcholine (LPC), and lysophosphatidic acid (LPA) were not significantly impacted in the absence of *Pf*PNPLA2, suggesting they might not be the direct products of the enzyme. The most striking change, however, was an extremely significant increase of phosphatidylglycerol (PG). PG is usually a low represented class of membrane PL that is also the substrate for the essential *de novo* synthesis of the mitochondrial-signature lipid class, cardiolipin. However, no change was observed in the levels of cardiolipin (CL) in the absence of *Pf*PNPLA2, again suggesting CL not being the product of the phospholipase. Interestingly, PG can also be the substrate of PLA2s to generate lysophosphoglycerol (LPG), or LBPA/bismonoacylglycerolphosphate (LBPA/BMP), of which both are important lipid precursor/intermediates also present in apicomplexa in low amounts (Hullin-Matsuda et al., 2007; Saric et al., 2016; Nolan et al., 2017; Amiar et al., 2020). LBPA/BMP can also possibly be made from cardiolipin but the usual substrate in mammalian cells is usually PG (Hullin-Matsuda et al., 2007). We thus sought to quantify the levels of LPG, LBPA, and PG, and determine their potential change in content upon the disruption of *Pf*PNPLA2 (**Figure 4B**). Our approach thus reveals that upon the loss of the enzyme, there was a significant accumulation of PG together with a very significant decrease of LBPA, whilst LPG levels were not significantly impacted (though slightly reduced). This significant decrease in LBPA was further confirmed by immunofluorescence (using an anti-LBPA antibody), which showed a significant decrease in fluorescence within the parasite when the protein expression was inhibited by GlcN treatment (**Figures 4C, D**). Taken together, the data strongly suggest that *Pf*PNPLA2 is responsible for the degradation of PG to form LBPA, which further induces PL homeostasis disruption (see hypothesis model in **Figure 5**).

## Discussion

Phospholipases contribute to the virulence of many pathogens, and more specifically to *Plasmodium* parasites (Flammersfeld et al., 2020). This family of proteins is pivotal for PL recycling, which direct a diversity of physiological roles via the regulation of lipid homeostasis for membrane synthesis but also through the control of key signaling pathways that govern host egress, secretion and developmental switching (Brancucci et al., 2017; Llinás, 2017; Flammersfeld et al., 2018; Wilson and Knoll, 2018; Flammersfeld et al., 2020; Asad et al., 2021). Knowing the importance of lipids, membrane biogenesis and homeostasis for the development and survival of *P. falciparum*, the enzymes manipulating lipids must play central essential parts. Phospholipases are such enzymes as they can hydrolyze membrane PL, either existing from the host or not, to enable the release of FA and LPL that can be used for parasite membrane homeostasis. Interestingly, they are ones of the rare metabolic genes located in the sub-telomeric region of *P. falciparum* genome, potentially favoring gene duplication as for *var* and *rifin* surface antigens, reflecting their putative importance for the parasite (Bethke et al., 2006). Their unusually high numbers compared to typical eukaryotes also reflects their importance for *P. falciparum* (Flammersfeld et al., 2018; Wilson and Knoll, 2018). Their exact roles for the parasite remain poorly understood but recent studies have started revealing differential localization and non-redundant and major functions such as hemozoin formation, parasite division or gametocyte formation (Singh et al., 2019; Flammersfeld et al., 2020; Asad et al., 2021; Sheokand et al., 2023). The few that have been investigated also show potential as drug targets (Yoo et al., 2020; Sheokand et al., 2023). To date however, the exact functions and mechanisms of most phospholipases remain to be unraveled.

As with PC and LPC, PL and lysoPL balances in the parasite can govern their cell development and are at the mercy of phospholipases (Brancucci et al., 2017; Flammersfeld et al., 2018). This study focused on a putative phospholipase enzyme containing a patatin-like phospholipase domain (**Figure 1A**). We generated an inducible knock-down line for *Pf*PNPLA2 (PF3D7_1358000) using the glmS-ribozyme system (Prommana et al., 2013). We revealed that *Pf*PNPLA2 only seems to be important for parasite replication in asexual parasites under nutrient stress, specifically when exogenous lipids are limited (**Figures 2C, D**). This seems to be in accordance to the essentiality data from a genome-wide saturation mutagenesis screen in *P. falciparum* (Zhang et al., 2018).

*Pf*PNPLA1 (PF3D7_0209100), has been previously identified as critical for gametocyte induction (Flammersfeld et al., 2020) and the process of gametocytogenesis itself (Singh et al., 2019; Yoo et al., 2020). *Pf*PNPLA2-HA-tagged parasites in this study have been able to prove the mitochondrial localization of the enzyme, although vicinity to the apicoplast is sometimes observed during schizont stages. This is supported by the predicted mitochondrial targeting sequence found in N-terminal end of the protein (Singh et al., 2019).

A putative dual localization in both mitochondrion and apicoplast is less likely but still possible as it is known that the two organelle are in close vicinity and share metabolic pathways, such as heme synthesis, between the two (Ralph et al., 2004; Botte et al., 2011). Currently there is no known shared lipid metabolism pathways between the two organelles, even if this could be occurring, notably for potential lipid exchange between them. The *T. gondii* homologue of *Pf*PNPLA2 (*Tg*PL2) does solely localize to the apicoplast, and is involved in apicoplast biogenesis, and parasite lipid homeostasis, more precisely by affecting levels of PC and LPC (Lévêque et al., 2017). The proximity to the mitochondria suggests that *Pf*PNPLA2 could be involved in mitochondrial membrane biogenesis through the generation of FA and lysoPL precursors. We hypothesized that this could be linked to cardiolipin synthesis as it only occurs at the mitochondrial level (Aho and Rostedt, 1973). The full cardiolipin synthetic pathway is yet to be characterized in *P. falciparum*, but in other eukaryotes, it requires PG as a substrate (Gallala and Sandhoff, 2011). Lipidomics revealed that PG was the most strikingly affected PL class in the absence of *Pf*PNPLA2, yet without significantly affecting cardiolipin levels. The disruption of the protein resulted in the accumulation of PG instead, unlike in the knockout of the *T. gondii* homologue of *Pf*PNPLA2, which displayed increased levels of PC (Lévêque et al., 2017). However, PG, and/or LBPA can be both degraded through the action of PLA2s to form LBPA (i.e. BMP-bis(monoacylglycerol)phosphate). In eukaryotes, LBPA is a lipid class normally involved in slow catabolism, such as degradation of lipid stores for energy production in nutrient poor conditions (Goursot et al., 2010). LBPA is negatively charged at the acidic pH of lysosomes, and these charges are central to its role in the degradation of lipids and membranes in the lysosome. In effect, these negative charges facilitate the adhesion of the soluble positively charged hydrolases and activator proteins (such as PLAs), thus allowing them to degrade the lipids at the interface of the inner membranes of the lysosome (Gallala and Sandhoff, 2011). Here, our results indicate that *Pf*PNPLA2 acts on the mitochondrial degradation of PG to form LBPA. Whether the synthesis of LBPA is important for blood stage parasite survival is an opened question.

Another important point is that the most significant growth phenotype occurred in low host lipid environment. We and others showed that host nutrient levels directly influence parasite metabolic activity, especially lipid metabolism, which can be rewired/adapted by the parasite upon host environment (Walsh et al., 2022; Brancucci et al., 2017; Mancio-Silva et al., 2017; Dass et al., 2021; Shunmugam et al., 2022). Indeed, *de novo* lipid synthesis and host scavenging activities can be used alternatively to compensate one another (Amiar et al., 2020; Dass et al., 2021). Host lipid scavenging constitutes the major source of parasite lipids and can be upregulated if *de novo* synthesis is insufficient. Scavenged lipids, specifically their FA moieties, are usually channeled towards lipid storages, usually lipid droplets, in the form of TAG, which can be timely mobilized for membrane biogenesis during parasite division (Dass et al., 2021; Sheokand et al., 2023). Here, we see that disrupting *Pf*PNPLA2 affects parasite growth only under low host lipid environment, indicating that the enzyme likely plays a role to boost lipid synthesis when scavenging is scarce (**Figure 5**). This is further correlated by the increase in C16:0 and C18:1 that are accumulating in TAG during schizont, and ring stages in the absence of *Pf*PNPLA2 in high host lipid content. This suggests that the parasite increases scavenging, and lipid storage, to compensate for the loss of *Pf*PNPLA2, as previously observed for other the loss of the *P. falciparum* lysophospholipase *Pf*LPL3 (Sheokand et al., 2023). This is further correlated by the additional accumulation of all neutral lipids, TAG, DAG and FFA during ring stages, reflecting the accumulation of scavenged material normally channeled through the storage lipid synthetic pathway, as previously observed (Dass et al., 2021; Sheokand et al., 2023).

One possible hypothesis (**Figure 5**) for the reduction of parasite population in the absence of *Pf*PNPLA2 under low host lipid content is that, similarly as in the *Pf*LPL3 mutant, the parasite does not have enough supply of FA by the degradation of its lipid substrate, in this case PG. This, in return, could prevent the parasite lipid synthesis machinery to form PLs for parasite membrane biogenesis during division, hence reducing progenies and overall parasitemia. This is further supported by the significant decrease of major PL classes PC, PE, PI and PS when *Pf*PNPLA2 is absent (**Figure 5**). A potential explanation is that the degradation of PG can provide FA moieties for the synthesis of major lipid classes when host lipid resources are scarce, hence enabling optimal progeny formation even under sub-optimal physiological conditions.

Taken together, our results point at the importance of *Pf*PNPLA2 to maintain proper parasite lipid homeostasis during blood stage replication, especially under low host nutrient content. It also points at the diversity and importance of the phospholipase activities that the parasite has developed and uses to maintain optimal asexual division, and likely for other life stages. The number and diversity of them advocate for importance, and probable redundancy as well. Antibodies raised in this study against native putative phospholipases, revealed differential localization of phospholipase suggesting a redundancy/varied functionality (**Figures 1C–G**). Phospholipase functional redundancy is well known and illustrated in *Listeria monocytogenes*, where mutation of a single phospholipase C resulted in moderate effects on mice infectivity and was severely enhanced when both were disrupted (Smith et al., 1995). This interplay of phospholipases could be extrapolated to malaria parasites, but further gene-knock out studies are needed to understand the essentiality of this family of proteins.

## Data availability statement

The raw data supporting the conclusions of this article will be made available by the authors, without undue reservation.

## Ethics statement

Ethical approval was not required for the studies on humans in accordance with the local legislation and institutional requirements because only commercially available established cell lines were used. Experiments for the generation of antisera in mice were approved by the animal welfare committees of the District Council of Cologne, Germany (ref. no. 84-02.05.30.12.097 TVA).

## Author contributions

All authors listed have made a substantial, direct, and intellectual contribution to the work and approved it for publication.
