# Supplementary material for: The patatin-like phospholipase PfPNPLA2 is involved in the mitochondrial degradation of phosphatidylglycerol during Plasmodium falciparum blood stage development
Source: Front Cell Infect Microbiol. 2023 Nov 20;13:997245. doi: 10.3389/fcimb.2023.997245 (PMC10711835; doi:10.3389/fcimb.2023.997245)
Supplement: Supplementary file 1 [file DataSheet_1.docx]

## SUPPLEMENTARY INFORMATION


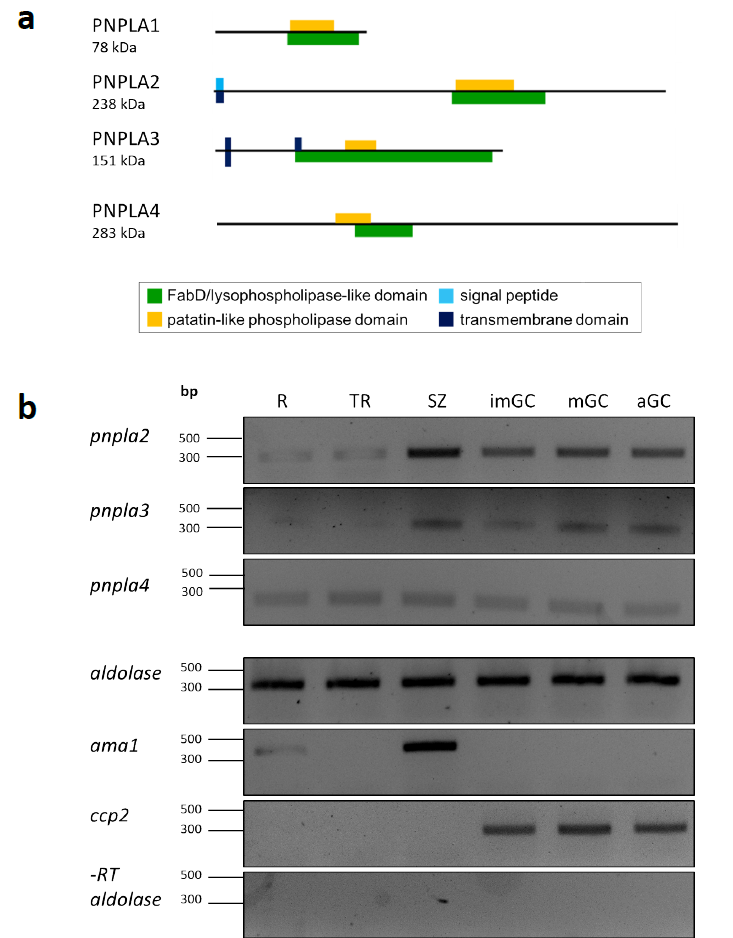


**Supplementary Figure 1: The PNPLA family of *P. falciparum*.** a) Schematic depicting the four members of the *P. falciparum* PNPLA family. b) Transcript expression of *Pf*PNPLA2-4 in the asexual and sexual blood stages. Diagnostic RT-PCR was used to amplify transcript of *pfpnpla2* (250 bp)*, pfpnpla3* (250 bp)*,* and *pfpnpla4* (250 bp) from cDNA generated from total RNA of rings (R), trophozoites (TR), schizonts (SZ), as well as immature gametocytes (imGC; stages II-IV), mature gametocytes (mGC; stage V) and activated gametocytes (aGC; 15’ p.a.). Transcript analysis of *ama1* (407 bp) and *ccp2* (286 bp) were used to demonstrate purity of the asexual and sexual blood stage samples. Transcript analysis of *aldolase* (378 bp) was used as loading control, RNA samples lacking reverse transcriptase (-RT) were used to prove the absence of gDNA. For transcript expression of *pfpnpla1*, see Flammersfeld et al., 2020.


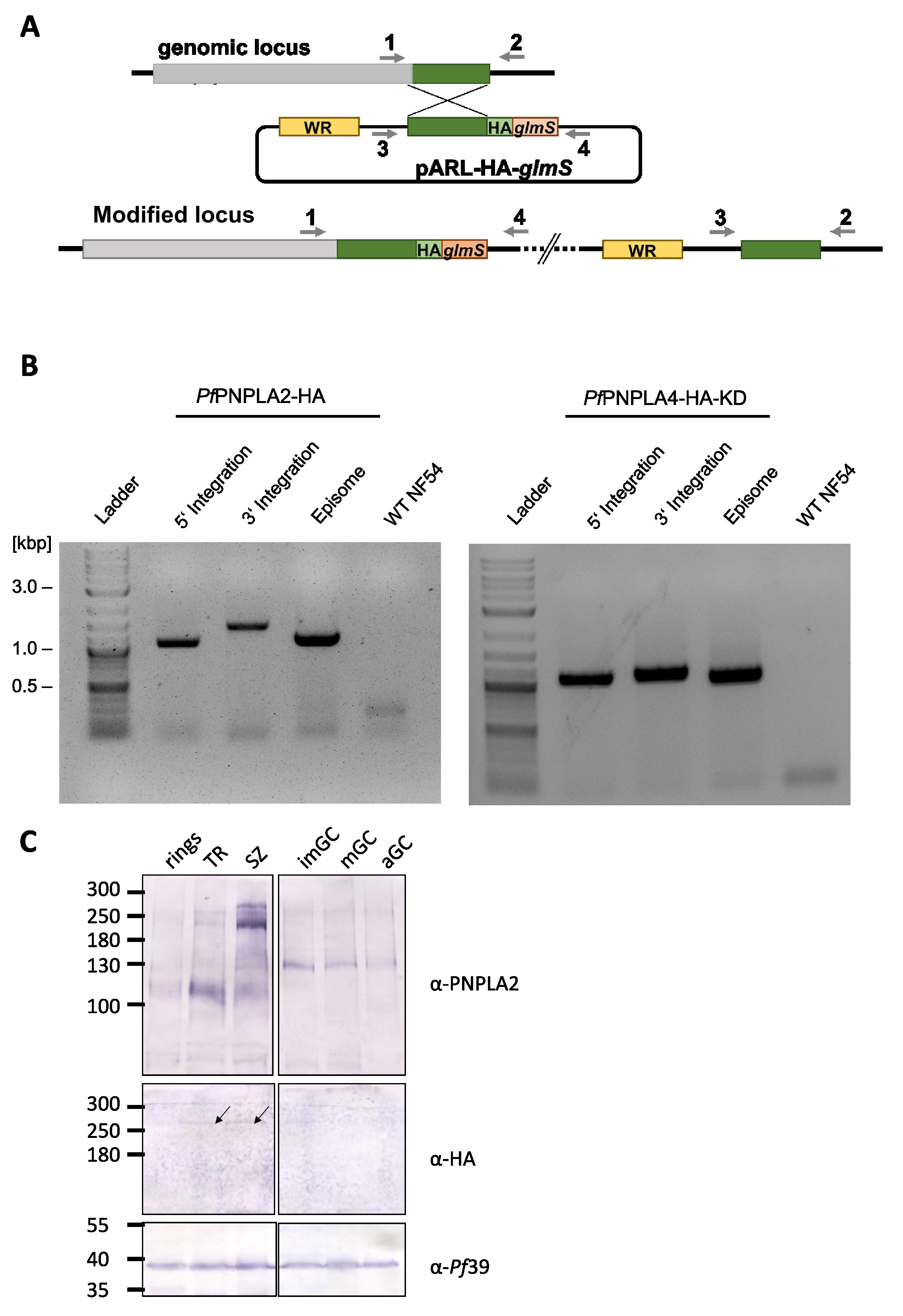


**Supplementary Figure 2: Generation of the *Pf*PNPLA2-HA-KD and *Pf*PNPLA4-HA-KD lines.** a) Schematic depicting the single-crossover homologous recombination strategy for the generation of the *Pf*PNPLA2-KD and *Pf*PNPLA4-KD lines. The coding region of *pfpnpla2* or *pfpnpla4* was fused at the 3’-region to an HA-encoding sequence followed by the *glmS*-ribozyme sequence. The numbered arrows indicate positions of primers used to confirm integration of the pARL-HA-*glmS* vector. WR, *hDHFR* gene conferring resistance to WR99210. b) Confirmation of gene locus integration of the pARL-HA-*glmS* vector. Diagnostic PCR using gDNA of the *Pf*PNPLA2-HA-KD and the *Pf*PNPLA4-HA-KD lines demonstrates successful 5′ (primers 1 and 4; 1,182 bp for *Pf*PNPLA2-HA-KD; 1,129 pb for the *Pf*PNPLA4-HA-KD) and 3′ (primers 2 und 3; 1,575 for *Pf*PNPLA2-HA-KD; 1,166 bp for the *Pf*PNPLA4-HA-KD) integration. As a control, amplification of WT NF54 gDNA was aimed (primers 1 and 2; 1,501 bp for the *Pf*PNPLA2-HA-KD; 1,205 bp for the *Pf*PNPLA4-HA-KD). Episomal DNA was further detected (primers 3 and 4; 1,250 bp for the *Pf*PNPLA2-HA-KD; 1,075 for the *Pf*PNPLA4-HA-KD).


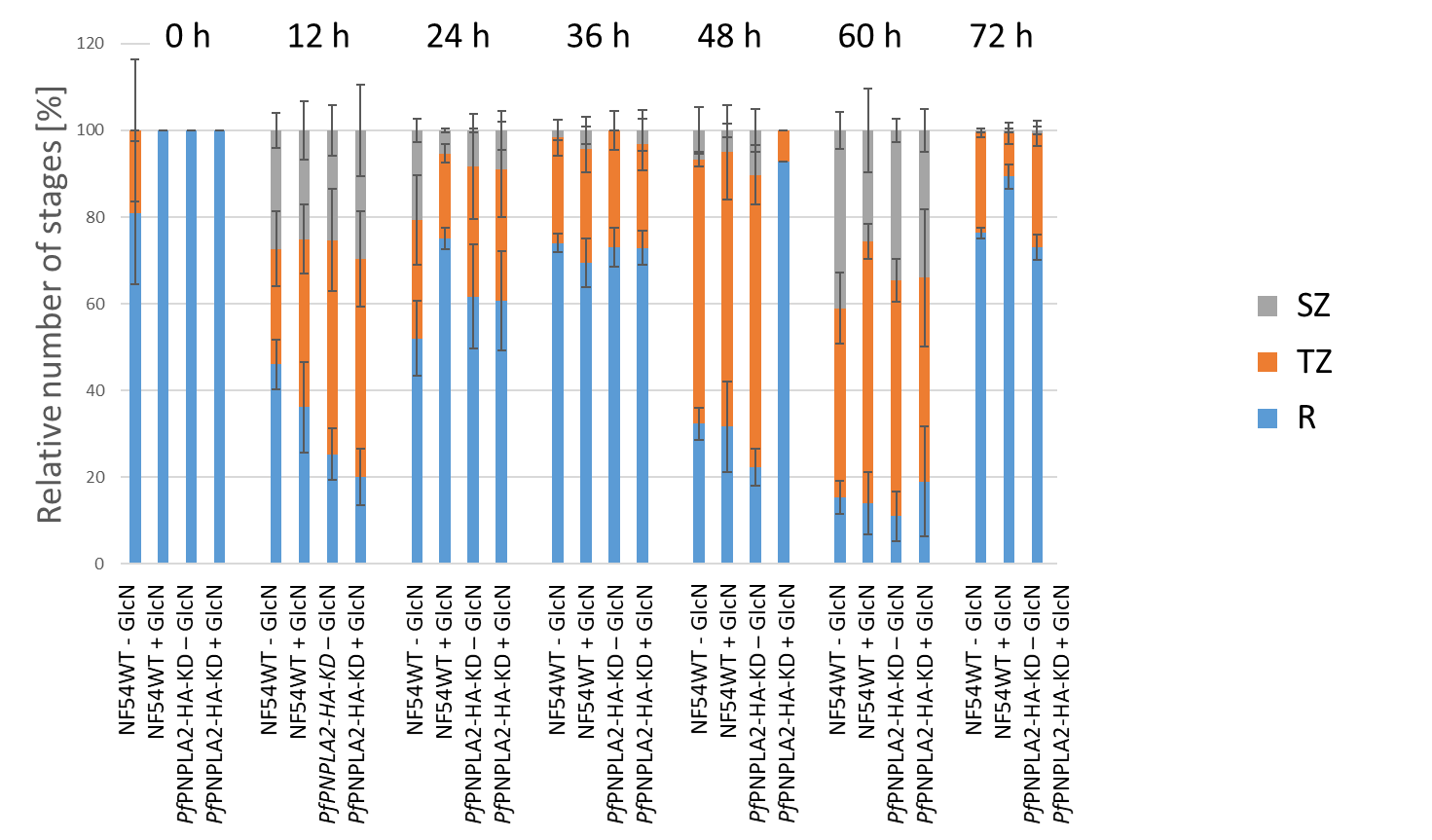


**Supplementary Figure 3: The effect of *Pf*PNPLA2-HA deficiency on asexual blood stage development.** Synchronized ring stage cultures of WT NF54 and the *Pf*PNPLA2-HA-KD parasite line were treated with 2.5 mM GlcN at a starting parasitemia of 0.25% and the cultures were maintained in cell culture medium with GlcN over a time-period of 72 h. Rings (R), trophozoites (TZ), and schizonts (SZ) were determined in a total number of 100 iRBCs in triplicate every 12 h via Giemsa-stained blood smears. Untreated cultures served as controls.


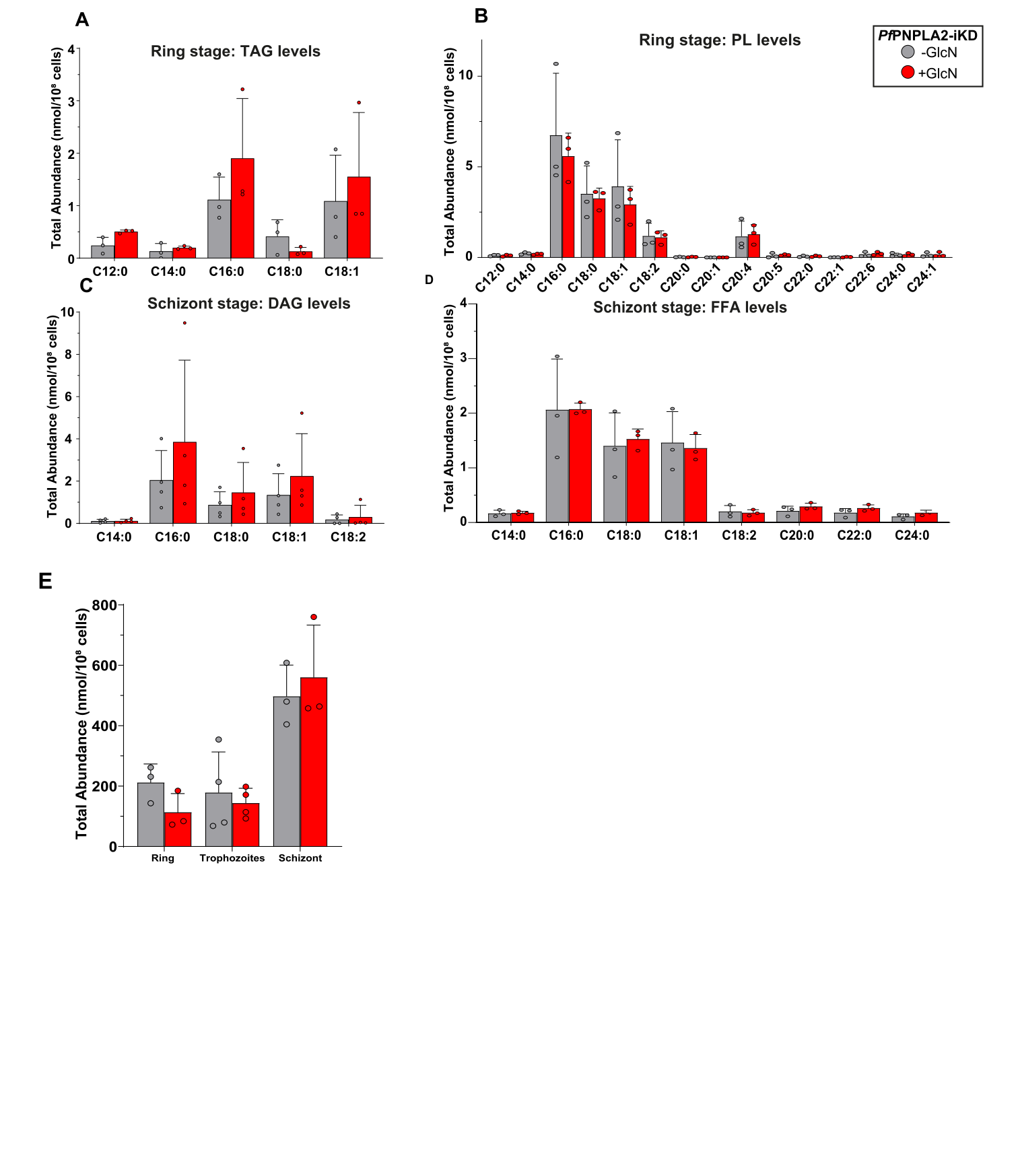


**Supplementary Figure 4: PfPNPLA2 depletion influences the lipidomic profile of asexual stage parasites.** Under the depletion of PfPNPLA2 (+GlcN, red) the lipid profile (in nmol/cell) of ring stage a) TAG and b) PL levels remain unaltered. The same is true for schizont stage c) DAG and d) FFA levels under depletion of the protein. e) No changes in Total lipid abundance were seen in any asexual stages of the parasite.
